# Supplementary material for: Unveiling the secrets of adeno-associated virus: novel high-throughput approaches for the quantification of multiple serotypes
Source: Mol Ther Methods Clin Dev. 2023 Sep 20;31:101118. doi: 10.1016/j.omtm.2023.101118 (PMC10562196; doi:10.1016/j.omtm.2023.101118)
Supplement: Document S1. Figure S1 and Table S1 [file mmc1.pdf]

OMTM, Volume *31*

Supplemental information

Unveiling the secrets of adeno-associated virus: novel high-throughput approaches for the quantification of multiple serotypes

Frederik Meierrieks, Ahmad Kour, Marvin Pätz, Karl Pflanz, Michael W. Wolff, and Andreas Pickl

Table S1: Cost estimation per sample for each method. The costs per data point are calculated based on the respective consumable costs and available sample positions. Expenses related to instrument costs and salaries were excluded from the estimation process. Consumable expenses for each method were derived from the information available on the German manufacturer's website. It should be noted that prices vary between countries and depending on the order quantity. Therefore, the table provides only a rough overview of the costs per data point.

	**Method**	**Price per data point [€]**
Genome titer	ndPCR (Qiagen)	3-4
	mapdPCR (Thermo Fisher)	6-7
	cdPCR (Stilla)	3-4
	ddPCR (Bio-Rad)	1-2
	qPCR (Bio-Rad)	1-2
Capsid titer	Capsid ELISA	8-9
	BLI (Octet®)	5-6
Transducing titer	Live-cell analysis (Incucyte® S3)	~1
	Flow cytometry (iQue Screener PLUS)	~1

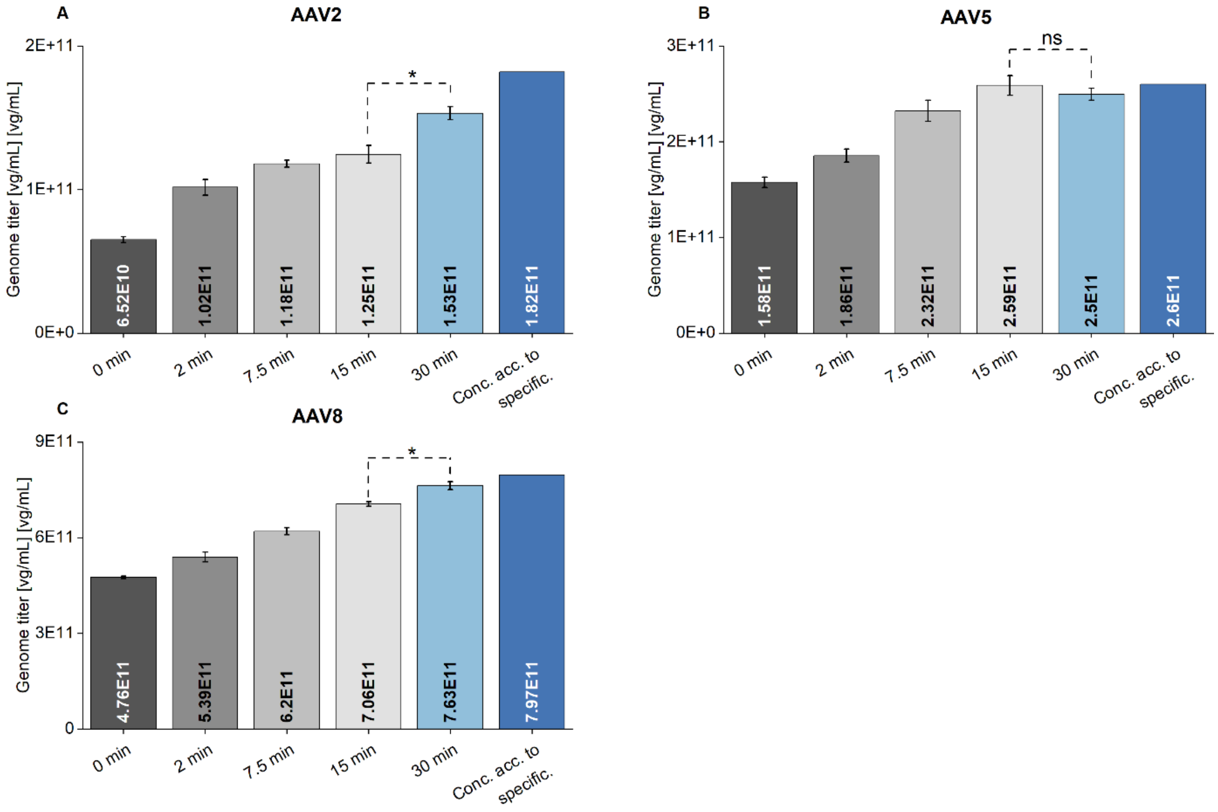

Figure S1: Impact of the heat incubation on AAV capsid disassembly. AAV2, AAV5, and AAV8 reference standard materials (AMSBIO Europe, Alkmaar, The Netherlands) were incubated at 95°C for different times prior to a 2 min initial denaturation at 95°C during PCR. The VG titers of AAV2 (**A**), AAV5 (**B**), and AAV8 (**C**) reference standard materials were determined by ndPCR (Qiagen) targeting the SV40 signal on the AAV genome. Error bars represent standard deviations of independent triplicate measurements. Statistical analysis was performed by unpaired t-test with a significance level of α=0.05. Statistically significant difference is denoted by an asterisk (*), whereas "ns" indicates no significant difference. Abbreviations: vg, viral genome.
