# Supplementary material for: Unveiling the secrets of adeno-associated virus: novel high-throughput approaches for the quantification of multiple serotypes
Source: Mol Ther Methods Clin Dev. 2023 Sep 20;31:101118. doi: 10.1016/j.omtm.2023.101118 (PMC10562196; doi:10.1016/j.omtm.2023.101118)
Supplement: Document S2. Article plus supplemental information [file mmc2.pdf]

Molecular Therapy
Methods & Clinical Development
Original Article

Unveiling the secrets of adeno-associated virus: novel high-throughput approaches for the quantification of multiple serotypes

Frederik Meierrieks,[1] Ahmad Kour,[1] Marvin Pätz,[2] Karl Pflanz,[2] Michael W. Wolff,[3,4,5] and Andreas Pickl[1,5]

[1]Lab Essentials Applications Development, Sartorius Lab Instruments GmbH & Co. KG, Otto-Brenner-Straße 20, 37079 Göttingen, Germany; [2]Lab Essentials Applications Development, Sartorius Stedim Biotech GmbH, August-Spindler-Straße 11, 37079 Göttingen, Germany; [3]Institute of Bioprocess Engineering and Pharmaceutical Technology, University of Applied Sciences Mittelhessen (THM), 35390 Giessen, Germany; [4]Fraunhofer Institute for Molecular Biology and Applied Ecology (IME), 35392 Giessen, Germany

Adeno-associated virus (AAV) vectors are among the most prominent viral vectors for *in vivo* gene therapy, and their investigation and development using high-throughput techniques have gained increasing interest. However, sample throughput remains a bottleneck in most analytical assays. In this study, we compared commonly used analytical methods for AAV genome titer, capsid titer, and transducing titer determination with advanced methods using AAV2, AAV5, and AAV8 as representative examples. For the determination of genomic titers, we evaluated the suitability of qPCR and four different digital PCR methods and assessed the respective advantages and limitations of each method. We found that both ELISA and bio-layer interferometry provide comparable capsid titers, with bio-layer interferometry reducing the workload and having a 2.8-fold higher linear measurement range. Determination of the transducing titer demonstrated that live-cell analysis required less manual effort compared with flow cytometry. Both techniques had a similar linear range of detection, and no statistically significant differences in transducing titers were observed. This study demonstrated that the use of advanced analytical methods provides faster and more robust results while simultaneously increasing sample throughput and reducing active bench work time.

INTRODUCTION

The pharmaceutical relevance of viruses includes their use as vaccines, oncolytic viruses, and gene therapy vectors.[1] Adeno-associated virus (AAV) is among the most promising viral vectors for gene therapy,[2,3] and currently, 350 clinical trials are ongoing using AAV as a vector.[4] Hemgenix, Luxturna, and Zolgensma are three AAV-based gene therapeutics that have already been approved by the U.S. Food and Drug Administration (FDA).

AAV was discovered in adenovirus (AdV) preparations in the mid-1960s by Atchison and colleagues.[5] It belongs to the Parvoviridae family and the genus of *Dependoparvovirus*.[6,7] AAV is a non-enveloped,[1] 4.7 kb single-stranded DNA (ssDNA) virus with an icosahedral capsid of about 25 nm in diameter.[8–10] To date, 13 distinct human AAV serotypes (AAV1–13) were discovered that are characterized by different capsid proteins and show tropisms for a diverse variety of cell and tissue types.[11–13] AAV2 is the most thoroughly investigated serotype.[14]

During the release of AAV products, but also during the manufacturing and purification process, it is essential to establish critical quality attributes (CQAs) to ensure product safety and quality. A CQA is defined as "a physical, chemical, biological, or microbiological property or characteristic that should be within an appropriate limit, range, or distribution to ensure the desired product quality."[15] In this study, we focused on the strength-related CQAs of AAV, which include viral genomic (VG) titer, capsid titer, and transducing titer.[16]

The VG titer is a measure of the number of vector genomes present in an AAV sample.[16] It is typically used for appropriate AAV dosing for preclinical and clinical phases.[17] VG titers are commonly determined by real-time qPCR or digital PCR (dPCR) methods targeting various regions of the AAV genome, including the inverted terminal repeats (ITRs), the simian virus 40 polyadenylation (SV40 poly[A]) signal, or the transgene of interest.[16]

qPCR and dPCR use either non-specific DNA-intercalating fluorophores such as SYBR Green or EvaGreen or specific fluorescence-labeled TaqMan probes.[18,19] dPCR is a technique similar to qPCR, with the distinction that in dPCR, individual DNA molecules are separated into compartments, enabling thousands of PCRs to be run in parallel. The presence or absence of a DNA molecule is identified through the detection of fluorescence, and the DNA copy number is calculated on the basis of the Poisson distribution.[17] Consequently, dPCR

Received 9 June 2023; accepted 14 September 2023;
https://doi.org/10.1016/j.omtm.2023.101118.

[5]These authors contributed equally

Correspondence: Frederik Meierrieks, Lab Essentials Applications Development, Sartorius Lab Instruments GmbH & Co. KG, Otto-Brenner-Straße 20, 37079 Göttingen, Germany.
E-mail: frederik.meierrieks@Sartorius.com

© 2023 The Author(s).
This is an open access article under the CC BY license (http://creativecommons.org/licenses/by/4.0/).

provides absolute quantification and eliminates the need for a standard curve,[20] minimizing variation in VG titer quantification.[21]

In addition to the VG titer, it is crucial to determine the capsid titer of an AAV sample or product, given that not all AAV particles contain a genome. The capsid titer represents the number of capsids, irrespective of their genomic content.[16,22]

Serotype-specific ELISA is the most widely used method for capsid titer quantification.[23] Bio-layer interferometry (BLI) is an alternative approach for capsid titer determination. BLI is a technique used to measure the interference pattern of white light reflected from a layer of immobilized proteins and an internal reference layer.[24] Biosensors that are coated with a ligand are used to detect binding of the analyte to the immobilized ligand, which in turn increases the layer thickness on the biosensor surface.[25] Aside from ELISA and BLI, alternative techniques for capsid titer quantification are size-exclusion chromatography coupled with multi-angle light scattering (SEC-MALS) or flow virometry.[22]

Because of the potential for mispackaged or fragmented genomes, the functionality of an AAV sample is not immediately evident from its VG titer. Moreover, harsh purification conditions may damage the capsid. As a result, it is essential to assess the potency of an AAV sample.

The transducing titer of AAV is commonly assessed using cell-based *in vitro* transduction assays, such as the 50% tissue culture infectious dose ($TCID_{50}$) assay,[26] infectious center assay,[26,27] or by flow-cytometry-based detection of fluorescent protein expression or fluorescent marked viral proteins.[21] The latter involves transduction of cells with AAV and analyzing them by flow cytometry after a designated period. This approach is applicable, for instance, to recombinant AAVs (rAAVs) that encode fluorescent proteins as transgenes or transgenes and viral proteins that can be detected using fluorescence-labeled antibodies.[28] Live-cell analysis is another potential method for determining the transducing titer, although it has not been reported for AAV so far. Like flow cytometry, live-cell analysis is based on the transduction of cells and subsequent excitation and detection of fluorescent or fluorescence-labeled proteins or probes.[29] Microscopic images of cells are captured before and after AAV transduction at user-defined intervals and can provide real-time information on cell physiology, such as confluence or transgene expression,[30,31] which in turn enables determination of the transducing titer.

In this study, AAV VG titers, capsid titers, and transducing titers were experimentally evaluated using qPCR, dPCR, ELISA, BLI, flow cytometry, and live-cell analysis. The main focus of this study was on handling, method comparability, sample throughput, and working range.

RESULTS

Genomic titer determination using qPCR and several dPCR techniques

A comparison of four dPCR methods, including crystal dPCR (cdPCR), nanoplate dPCR (ndPCR), droplet dPCR (ddPCR), and microfluidic array plate dPCR (mapdPCR), as well as a qPCR method to determine the VG titer of AAV2, AAV5, and AAV8 reference standard materials (RSM) was conducted. The assay developed for this study used a duplex technique targeting the SV40 and ITR region. VG titers resulting from the different PCR methods are displayed in Figure 1, while Table 1 presents the corresponding relative SDs and recovery values. The recovery rates are based on the specified qPCR-determined VG titers of the respective RSM.

For AAV2-SV40 (Figure 1A), the VG titers obtained from dPCR techniques exhibited significant variations ($\alpha = 0.05$, analysis of variance [ANOVA]), with values ranging from 1.14×10^{11} to 1.80×10^{11} viral genomes (vg)/mL and corresponding recovery rates of 62.7%–98.9%. In contrast, upon comparison with qPCR, which yielded a recovery rate of 103.1% at 1.82×10^{11} vg/mL, no significant differences ($\alpha = 0.05$, ANOVA) were observed. In the case of AAV5-SV40 (Figure 1B), no significant differences ($\alpha = 0.05$, ANOVA) were observed both between the dPCR assays and in comparison with qPCR, with a recovery of 98%–117.7% and VG titers of 2.55×10^{11} to 3.06×10^{11} vg/mL. Regarding AAV8-SV40 (Figure 1C), the values obtained from different dPCR methods were significantly different, but no significant difference was found when comparing all dPCR methods with qPCR ($\alpha = 0.05$, ANOVA). The dPCR methods resulted in VG titers of 7.70×10^{11} to 1.06×10^{12} vg/mL, with a corresponding recovery of 96.6%–133.3%, while qPCR yielded values of 1.04×10^{12} vg/mL, with a recovery rate of 130.7%. As shown in Figures 1D–1F and Table 1, the ITR VG titers determined by the different dPCR methods were significantly different ($\alpha = 0.05$, ANOVA) for AAV2 and AAV8, with recovery rates of 88.5%–136.7% and 131.5%–176.5%, respectively. Insignificant differences ($\alpha = 0.05$, ANOVA) were found for AAV5, and the recovery rate ranged from 149.3% to 171.7%. The ITR region qPCR results were not included in Figure 1, as the recovery rates for AAV2, AAV5, and AAV8 were between 1,450% and 1,957.8%, as presented in Table 1.

After genome titer determination of AAV RSM, the impact of the sample matrix on the different dPCR methods and qPCR was investigated. Clarified crude cell lysate samples from two different production processes (referred to “batch A” and “batch B”) were analyzed, and the results are illustrated in Figure 2A for AAV8 and the SV40 signal. The term “clarified crude cell lysate” refers to samples that underwent only minimal purification and were clarified solely by centrifugation. The VG titers of AAV8 batch A determined using dPCR methods ranged from 2.53×10^{10} to 3.68×10^{10} vg/mL, whereas qPCR yielded a genome titer of 5.00×10^{10} vg/mL. For AAV8 batch B, the VG titer determined by qPCR was 4.23×10^{10} vg/mL, while the dPCR methods resulted in genome titers of 1.65×10^{10} to 2.12×10^{10} vg/mL. Significant differences ($\alpha = 0.05$, ANOVA) were observed in both AAV8 batch A and AAV8 batch B among the dPCR methods but also compared with qPCR, with qPCR yielding 1.4- to 2.6-fold higher genome titers than dPCR. Similar trends were found for the ITR region and AAV2 and AAV5 (data not shown). The handling time of the various PCR methods was examined and normalized to a quantity of 16 samples (Figure 2B). The active working time

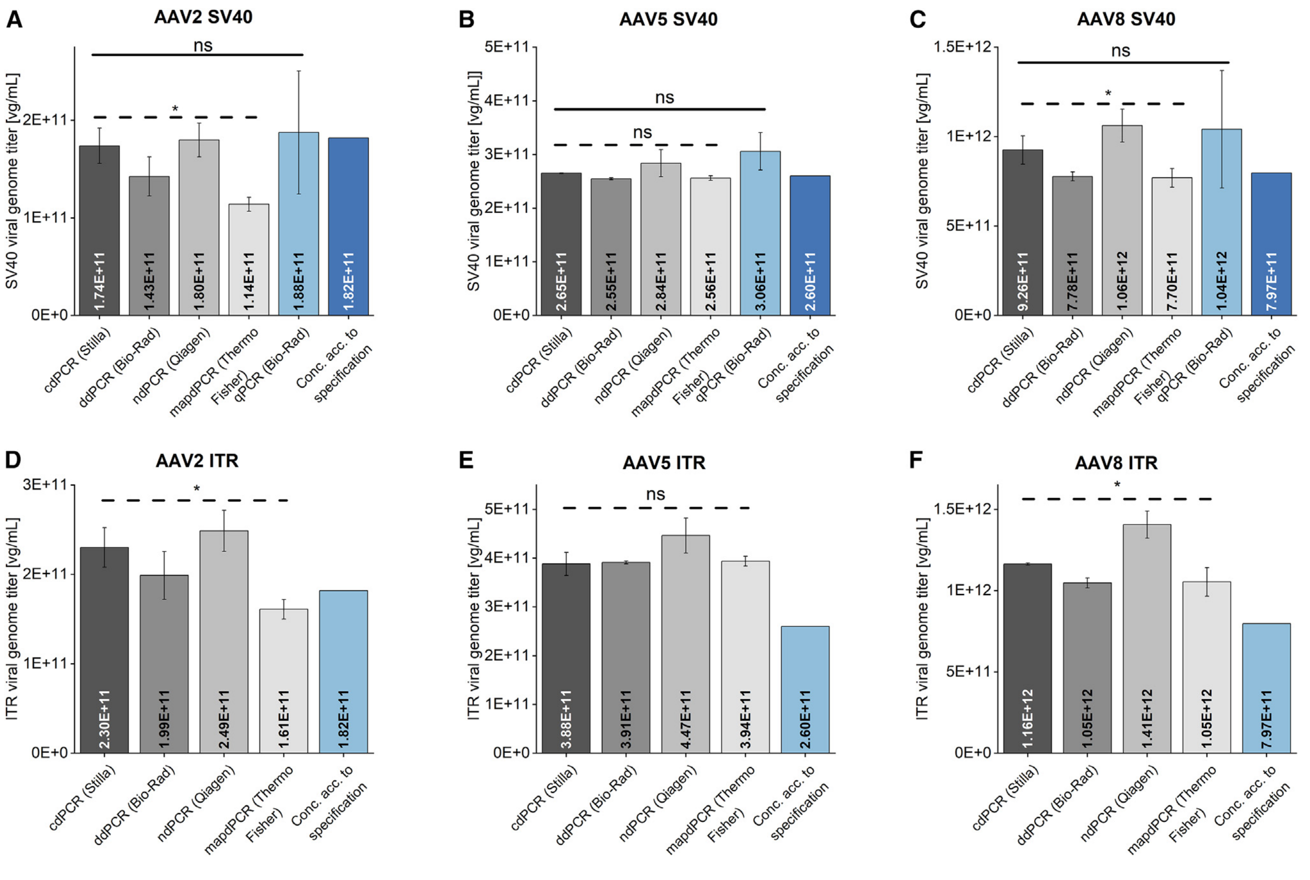

Figure 1. Genomic titers of different dPCR and qPCR methods

VG titers of AAV2, AAV5, and AAV8 reference standard materials (AMSBIO Europe, Alkmaar, the Netherlands) were determined by cdPCR, ndPCR, ddPCR, mapdPCR, and qPCR in a duplex assay targeting both the SV40 and ITR region on the AAV genome. The VG titers for either the SV40 or the ITR region are displayed for AAV2 (A and D), AAV5 (B and E), and AAV8 (C and F). Error bars represent SDs of independent triplicate measurements. Statistical analysis was performed using one-factor ANOVA with a significance level of $\alpha = 0.05$. Statistically significant difference is denoted by an asterisk, and "ns" indicates no significant difference. vg, viral genome; SV40, simian virus 40, ITR, inverted terminal repeats.

involves manual handling steps, such as pipetting, whereas the passive working time refers to waiting times, such as incubation, imaging, or measurement times. Preparation of 16 samples and their transfer to the respective plate or chip was identical for all methods and took approximately 1.5 h. The PCR cycling time was similar for all methods at about 2 h. These factors did not differ among the methods and were therefore excluded from the active and passive working time analysis. All methods included an active working time. For cdPCR, wiping the ruby chip with an anti-static cloth before imaging took 2 min, while sealing of the ndPCR and ddPCR plates required 2 and 3 min, respectively. In addition, mapdPCR required the use of an isolation buffer and the application of seals, which combined accounted for an active working time of 10 min. As qPCR involved the preparation of a plasmid standard in addition to sample preparation, an additional active working time of 15 min was required. The cdPCR, ddPCR, and qPCR methods also required passive working times of 12, 22.5, and 40 min, respectively. For cdPCR, this entailed imaging of the ruby chips, whereas ddPCR required droplet generation and reading of the droplets. In the case of qPCR, the passive working time involved enzymatic linearization digestion of the plasmid and subsequent inactivation of the enzyme. As the use of Proteinase K during sample preparation is a time-consuming step, we further investigated whether a heat incubation step is sufficient for capsid lysis of AAV2, AAV5, and AAV8 (see Figure 3). The achieved VG titers of samples with and without Proteinase K digest showed variation of less than 10% among one another and no significant differences ($\alpha = 0.05$, unpaired t test). To investigate another potential time-saving approach, we examined the required incubation time at 95°C during sample preparation (data shown in Figure S1). For AAV5, no statistically significant differences ($\alpha = 0.05$, unpaired t test) in VG titers were observed between a 15 min and a 30 min incubation period. However, significant differences were found for AAV2 and AAV8, with values differing by 19% and 8%, respectively.

Capsid titer determination using BLI and ELISA

The capsid titer was determined by ELISA and BLI. To identify the linear range of both methods, 2-fold and 5-fold serially diluted AAV8 samples were analyzed using capsid ELISA and BLI,

Table 1. Overview of the relative SDs and genomic titer recoveries of different dPCR and qPCR methods

		cdPCR (Stilla)		ddPCR (Bio-Rad)		ndPCR (Qiagen)		mapdPCR (Thermo Fisher Scientific)		qPCR (Bio-Rad)	
		CV	Recovery	CV	Recovery	CV	Recovery	CV	Recovery	CV	Recovery
	Target	(%)	(%)	(%)	(%)	(%)	(%)	(%)	(%)	(%)	(%)
AAV2	SV40	10.4	95.6	14	78.3	9.6	98.9	6.2	62.7	33.6	103.1
	ITR	9.6	126.5	13.5	109.3	9.2	136.7	6.8	88.5	43.3	1668.1
AAV5	SV40	0.2	102.1	0.8	98	8.9	109.2	1.7	98.5	11.4	117.7
	ITR	6.1	149.3	0.8	150.5	8.0	171.7	2.6	151.4	68.6	1450
AAV8	SV40	8.6	116.1	3.2	97.6	8.6	133.3	6.8	96.6	31.5	130.7
	ITR	0.5	146.0	2.9	131.5	5.9	176.5	8.4	132.3	43.5	1957.8

VG titers of AAV2, AAV5, and AAV8 RSM (AMSBIO Europe) were measured by cdPCR, ndPCR, ddPCR, mapdPCR, and qPCR as a duplex assay targeting both the SV40 and ITR region on the AAV genome. The recovery values were calculated with respect to the specified VG titer. Experiments were performed as independent triplicate measurements. CV, coefficient of variation; SV40, simian virus 40, ITR, inverted terminal repeats.

respectively. The resulting data in Figures 4A and 4B showed a linear dependence for ELISA between a capsid titer of 7.66×10^6 and 2.45×10^8 capsids (c)/mL, with an R^2 value of 0.99, and for BLI between 8.64×10^8 and 1.35×10^{13} c/mL, with an R^2 value of 0.99.

Following the determination of the linear range of ELISA and BLI, the capsid titers of AAV2, AAV5, and AAV8 samples from two different upstream processes were analyzed and are shown in Figure 4C. For AAV2 batch A and AAV2 batch B, ELISA yielded capsid titers of 1.55×10^{11} and 6.60×10^{12} c/mL, respectively. The corresponding capsid titers determined by BLI for AAV2 batch A and AAV2 batch B showed no significant differences ($\alpha = 0.05$, unpaired t test) from ELISA titers with 1.43×10^{11} and 4.14×10^{12} c/mL. For AAV5 batch A and AAV5 batch B, capsid titers determined by ELISA were 2.80×10^{12} and 3.83×10^{12} c/mL, respectively. Comparatively, BLI resulted in capsid titers of 3.54×10^{12} and 4.30×10^{12} c/mL. The differences in capsid titers of AAV5 batch B were statistically significant ($\alpha = 0.05$, unpaired t test). Similar results were obtained for AAV8 batch A, with ELISA and BLI yielding statistically significantly different ($\alpha = 0.05$, unpaired t test) capsid titers of 5.65×10^{12} and 6.32×10^{12} c/mL, respectively. The ELISA and BLI capsid titers of AAV8 batch B resulted in 7.83×10^{12} and 1.02×10^{13} c/mL, respectively.

The handling time of the two methods was analyzed for a size of 16 samples. Figure 4D illustrates the individual active and passive working times for both methods. The time required for sample preparation and transfer to the well plates was excluded from the analysis, as these steps did not differ among the methods. Emphasis was placed on distinguishing aspects of the two methods. The ELISA method required an active working time of 42 min, which included three wash steps and the addition of anti-AAV biotin conjugate, streptavidin peroxidase conjugate, substrate (tetramethylbenzidine), and stop solution (sulfuric acid). The passive working time of 86 min included four incubation steps of 20 min each and the reading of the 96-well plate in a microplate reader. In contrast, BLI required an active working time of 10 min, which included the addition of an assay, regeneration, and neutralization buffer to the 96-well plate. The passive working time of the BLI involved the measurement of the individual rows of the 96-well plate. Measurement of a single row of the 96-well plate takes 15 min plus subsequent washing and regeneration of the AAVX biosensors. Thus, the measurement of 16 samples and two rows of standard samples each resulted in a passive working time of 72.3 min.

Transducing titer determination using live-cell analysis and flow cytometry

Determination of the transducing titer was performed using live-cell analysis and flow cytometry of HEK293T cells transduced with AAV2. For the determination of the transducing titer using live-cell analysis with the Incucyte S3, it was necessary to define parameters for the phase contrast and to specify analysis masks for the confluent and green fluorescent areas. The defined analysis masks successfully detected both confluence and GFP expression, as illustrated in Figure 5.

In order to determine the linear range of the live-cell analysis and flow cytometry assays, HEK293T cells were transduced with a serially diluted AAV2 sample. The resulting data in Figure 6A showed a linear dependence, with an R^2 value of 0.99 for live-cell analysis and an R^2 value of 0.95 for flow cytometry with a multiplicity of infection (MOI) range of 50–450 vg/cell. According to the linear range obtained, the lower and upper limits of detection for live-cell analysis were determined to correspond to a percentage of GFP-positive cells of 19.6% and 59.6%, respectively. Similarly, the lower and upper limits of detection for the flow-cytometry-based protocol corresponded to a percentage of GFP-positive cells of 19% and 59%, respectively.

Following the determination of the linear range of both assays, the transducing titer of an AAV2 sample was analyzed for dilutions within the respective linear range (Figure 6B). Using live-cell analysis, the transducing titer was found to be 1.49×10^8 transducing units (TU)/mL. The transducing titer determined by flow cytometry demonstrated no significant differences ($\alpha = 0.05$, unpaired t test) at a concentration of 1.54×10^8 TU/mL. The handling time for

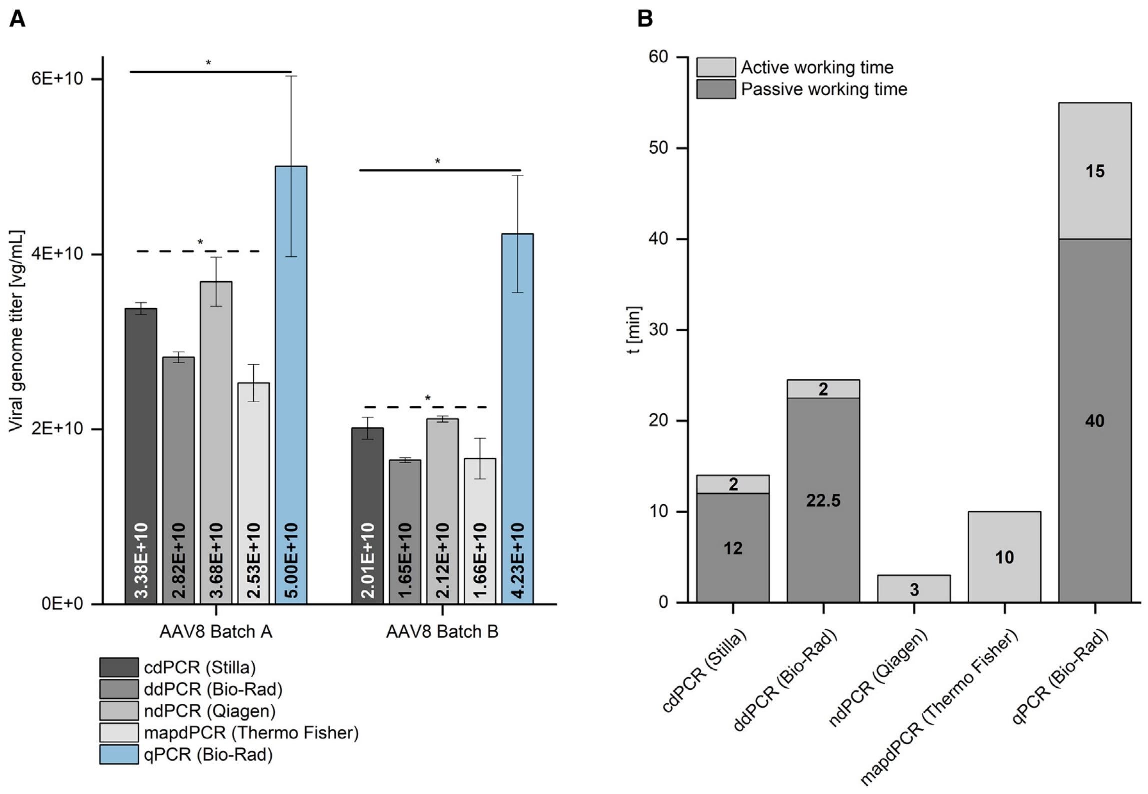

Figure 2. Impact of the sample matrix and working time of different dPCR and qPCR methods

VG titers of AAV samples obtained from two distinct upstream processes were determined by cdPCR, ndPCR, ddPCR, mapdPCR, and qPCR. Results are shown for AAV8 and the SV40 signal (A). In addition, the active as well as the passive working time for different dPCR and qPCR methods for the processing of 16 samples was analyzed (B). The active working time involves manual handling steps, such as pipetting and handling of consumables, whereas the passive working time refers to waiting times, such as incubation or imaging times. Preparation of 16 samples and their transfer to the respective plate or chip was identical for all methods and took approximately 1.5 h. Cycling time was similar for all methods at about 2 h. Therefore, these steps were excluded from the active and passive working time analysis. Error bars represent SDs of independent triplicate measurements. Values depicted were subjected to one-factor ANOVA with a significance level of α = 0.05; significant differences are represented by an asterisk. vg, viral genome; SV40, simian virus 40; t, time; Batch A and Batch B, respective upstream process.

both techniques was analyzed, and the individual active and passive working times were determined and are presented in Figure 6C. As the steps of cell seeding and cultivation, transduction, and media exchange did not differ between the two methods, these steps were excluded from the analysis. For live-cell analysis, no additional active steps were required beyond those already mentioned. Live-cell analysis solely involved a passive scan step of 12 min for the 96-well plate. Conversely, the flow-cytometry-based protocol required an active working time of 52 min, which consisted of several sequential steps. These steps included a 12 min trypsinization, a 10 min cell washing, 15 min cell fixation, and another 10 min cell washing step, followed by a 5 min resuspension of the cells. The passive working time of 40 min involved 30 min of purging time, as well as the calibration of the lasers and a 10 min measurement of the 96-well plate.

DISCUSSION

AAVs are currently among the leading vectors for *in vivo* gene therapy,[32] and enhancing their development using high-throughput screening approaches is of great interest. However, sample throughput is a bottleneck in most existing analytical methods.[22] Therefore, the objective of this study was to compare traditional AAV analytical methods with more advanced methods that enable higher sample throughput, leading to improved process comprehension and insight. A comprehensive table outlining the costs per sample for each respective method is also included in the supplemental material (Table S1). We evaluated the various analytical techniques using different AAV serotypes, as they are known to vary in attained titers, full/empty ratios, aggregation, and adsorption characteristics.[33]

A comparison of genomic titer determination using qPCR and several dPCR techniques

The determination of the VG titers of AAV2, AAV5, and AAV8, presented in Figure 1 and Table 1, resulted in varying outcomes when using the two targets SV40 and ITR. The results for the ITR region were higher by up to 57% than those for the SV40 signal. In contrast to AAV5, the comparison of dPCR methods among each other showed significant differences (α = 0.05, ANOVA) for AAV2 and AAV8 for the SV40 as well as for the ITR region. However, these differences

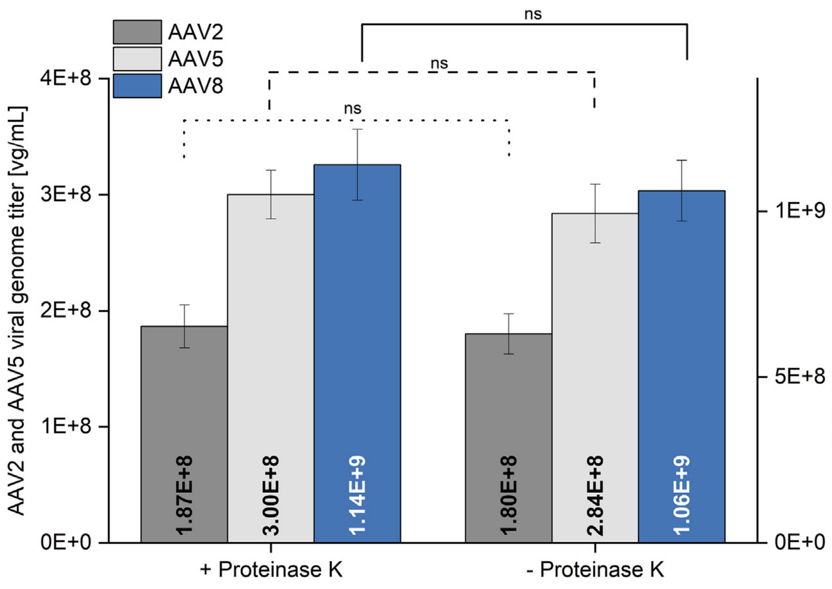

Figure 3. Sample preparation for capsid lysis

Serially diluted AAV2, AAV5, and AAV8 samples were lysed either by a Proteinase K digest or by heat incubation. Resulting VG titers are presented exemplifying the SV40 ndPCR on the QIAcuity One (Qiagen). Error bars represent SDs of independent triplicate measurements. VG titers were analyzed using an unpaired t test with a significance level of $\alpha = 0.05$, which is indicated by an asterisk. vg, viral genome; SV40, simian virus 40; +, with; -, without.

were rather small with an overall recovery of 78.3%–133.3% and 109%–176.5%, respectively (when AAV2 mapdPCR was excluded). These minor differences are expected, as the methods rely on different functional principles. While ddPCR and cdPCR are droplet-based methods, ndPCR and mapdPCR are based on partitioning on microfluidic plates, which might affect the distribution of the DNA template during dPCR. The increased ITR recovery values might be associated with truncated genomes.[21] Furuta-Hanawa et al.[34] also observed 50% higher ITR VG titers compared with those of SV40. The truncated genomes may have resulted from various factors, such as DNA extraction, the presence of a proportion of non-intact vector plasmids during AAV production, or defective packaging by HEK293 cells.[34] Genome packaging starts at the ITR region,[32] which occurs twice on the AAV genome. Consequently, mispackaged and truncated genomes are more likely to contain at least one ITR sequence than an SV40 sequence, which only appears once on the AAV genome. Although the ITR are typically considered conserved regions between most rAAV serotypes, we recommended to determine the VG titer on the basis of the transgene or other centrally located regions on the rAAV genome.

Despite multiple rounds of sample preparation and measurement, the recovery values of AAV2 mapdPCR (Table 1) for both SV40 (62.7%) and ITR (88.5%) were lower than those of the other dPCR methods. This phenomenon is not specific to the method, as it was not observed for AAV5 and AAV8. Inefficient singulation of the AAV2 genome during compartmentalization of mapdPCR could be a possible explanation for this discrepancy. As depicted in Figure 1, qPCR showed a relative SD of up to 69%, whereas the highest relative SD observed for all dPCR methods was $\leq 20\%$ (ndPCR). A possible explanation for the high SD observed with qPCR could be due to inconsistent amplification efficiency during exponential template amplification. As qPCR does not rely on endpoint measurements, the effect of such inconsistencies is stronger than in dPCR. Consequently, qPCR generates results with greater variability than dPCR.

AAV2, AAV5, and AAV8 VG titers determined by qPCR exhibited notable differences between the SV40 and ITR regions (Figure 1, Table 1). SV40-qPCR, with a recovery of 103%–130%, showed no significant differences ($\alpha = 0.05$, ANOVA) from the values determined using dPCR. In contrast, ITR-qPCR revealed differences from the VG titers determined using dPCR with recovery values of 1,450%–1,958%. As reported by D'Costa et al.[35] and Furuta-Hanawa et al.,[34] this strong overdetermination by a factor of up to 20 can be attributed to the double-stranded (ds) plasmid DNA (pDNA) used as the qPCR standard. The absence of the ITR region as free ends on the linearized ds-pDNA standard used in this study, in contrast to the rAAV ssDNA genome, complicates the denaturation of ITR in the ds-pDNA and consequently also primer annealing during qPCR. This reduces the actual pDNA copy number and thus artificially increases the genome titer of the ssDNA rAAV genome, in which the ITR are present as free ends.

Effect of sample matrix and working time analysis of qPCR and different dPCR methods

Genome titer determination of clarified crude cell lysate samples, as depicted in Figure 2A, revealed that qPCR resulted in genome titers higher by a factor of 1.4–2.6 compared with dPCR methods. Previously, as demonstrated in Figure 1 and Table 1, we found that genome titer values obtained by SV40-qPCR were comparable with those of SV40-dPCR and are also within the anticipated concentration range. Hence, in clarified crude cell lysate samples, qPCR appears to be more susceptible to interference from enhancers and inhibitors originating from the sample matrix than dPCR, as also reported by Cankar et al.[36] In addition, dPCR is known for its robustness toward the sample matrix.[16] Although the literature describes the need for Proteinase K treatment for capsid lysis in some protocols,[37] in other cases, no Proteinase K treatment is necessary.[18] AAV serotypes differ in their capsid stability depending on the composition of their capsid proteins, with AAV2 being the least stable and AAV5 the most stable serotype,[38] having a capsid melting temperature exceeding 90°C.[39] As shown in Figure 3, no significant differences ($\alpha = 0.05$, unpaired t test) occurred between the genome titers of AAV2, AAV5, and

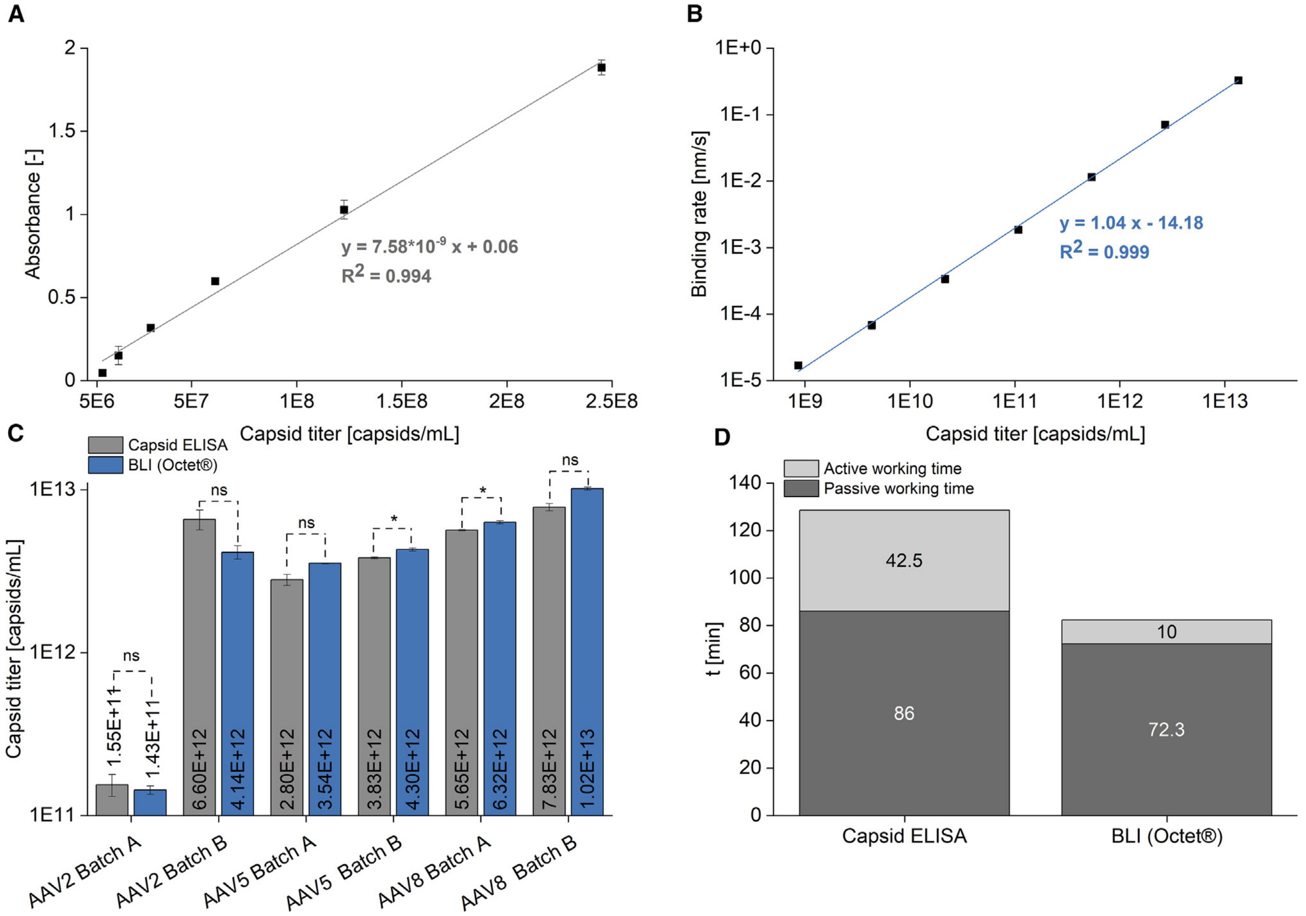

Figure 4. Capsid titer determination and handling times of capsid ELISA and BLI

A correlation between capsid titer and absorbance, as well as between capsid titer and binding rate, for ELISA (A) and BLI (B), is shown for AAV8. Furthermore, the determined capsid titers for AAV2, AAV5, and AAV8 samples, obtained from two distinct upstream processes, within the linear range by both methods are presented (C). In addition, the active as well as the passive working time for both techniques for the processing of 16 samples is shown (D). The active working time involves manual handling steps, such as pipetting, whereas the passive working time refers to waiting times, such as incubation or measurement times. Preparation of 16 samples and their transfer to the respective 96-well plates was identical for both methods and took approximately 1 h. Therefore, this step was excluded from the active working time analysis. Error bars represent SDs of replicate measurements (ELISA, n = 2; BLI, n = 3). Capsid titers were analyzed using an unpaired t test with a significance level of $\alpha = 0.05$. Significant differences are represented by an asterisk, and "ns" denotes no significant difference. Batch A and batch B, respective upstream process; t, time.

AAV8 samples when capsid lysis was performed with and without Proteinase K treatment. We demonstrated that no additional Proteinase K digestion was necessary for AAV2, AAV5, and AAV8 which corresponds to a time saving of 1 h. Furthermore, an additional experiment was performed to investigate the required pre-incubation time at 95°C during sample preparation (data presented in Figure S1). Considering that the majority of process-derived samples contain nucleases, a minimum pre-incubation of 15 min at 95°C is needed for proper inactivation of the nucleases before adding primers and other reagents to the sample. Our findings indicate that for AAV2 and AAV8, a pre-incubation period of 30 min is necessary to achieve adequate capsid disassembly. However, in the case of AAV5, a pre-incubation time of 15 min might be sufficient. To ensure a standardized protocol applicable to all serotypes, we recommend a pre-incubation period of at least 30 min for all process-derived samples.

When analyzing the handling times in Figure 2, we excluded the time required for sample preparation, transfer onto the plates/chips, and PCR cycling, as it was identical across all PCR methods. We observed that the additional active working time for all dPCR methods was ≤ 10 min, while qPCR required the longest active working time of 15 min because of the need for a plasmid standard restriction digestion step. In addition, qPCR revealed the highest additional passive working time of 40 min, which includes the time needed for restriction digest. Among the dPCR methods, only cdPCR and ddPCR had a passive working time of 12 and 22.5 min, respectively. This is attributed to the absence of fully integrated systems in the latter two methods, unlike ndPCR and mapdPCR, which use fully integrated systems. However, ddPCR may also be performed in a fully integrated system, such as the QX ONE ddPCR System (Bio-Rad). Overall, the dPCR methods differed only slightly in handling.

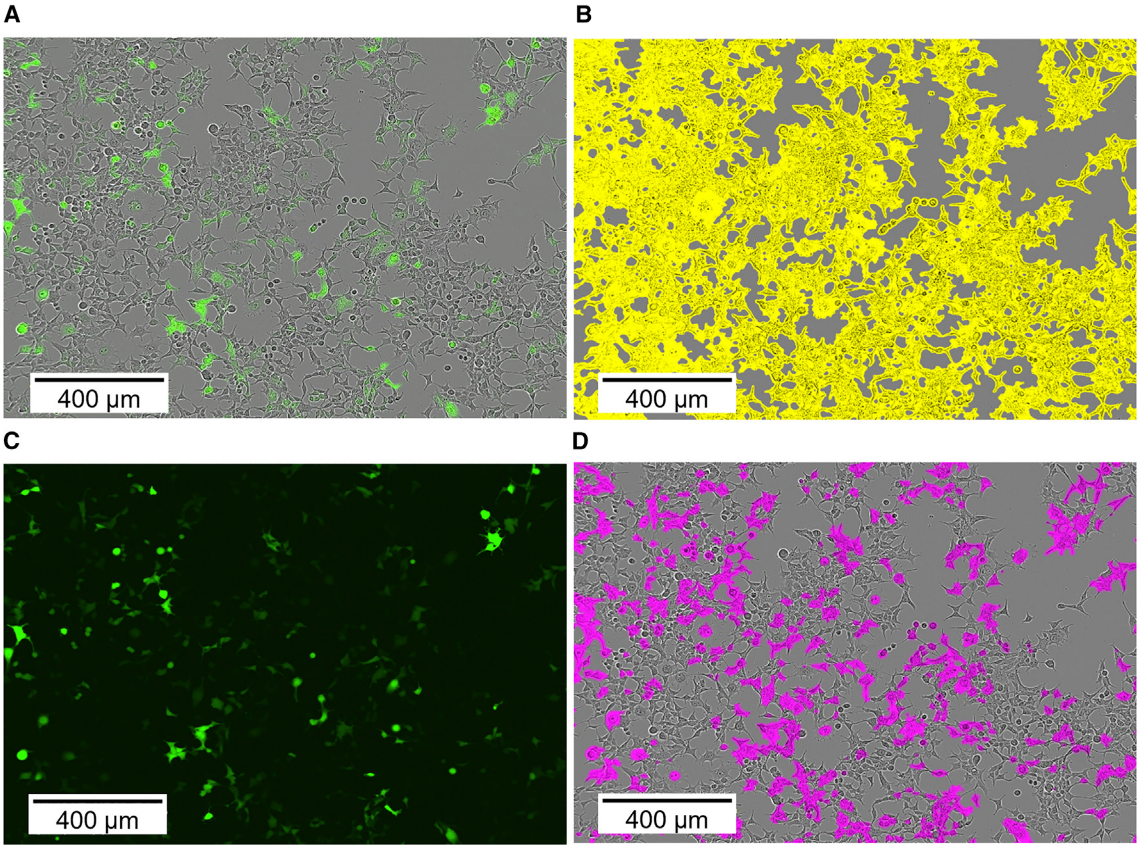

Figure 5. Analysis masks of live-cell analysis used to calculate the transducing titer

HEK293T cells were transduced with AAV2 batch A and GFP expression was analyzed 48 h post-transfection. Images were taken at 10× magnification. The phase contrast channel combined with the green fluorescence channel (A), and the phase contrast channel merged with the confluent area detection mask in yellow (B) are shown. Furthermore, the green fluorescence channel (C), and the phase contrast channel combined with the green fluorescence detection mask in pink (D) are presented.

Additionally, dPCR is an absolute quantification method, that does not require a standard curve and consequently eliminates the need for preparation of said standard. This leads to a time saving of more than 30 min, compared with qPCR.

Comparative studies have already been performed between qPCR and ddPCR for determination of the AAV VG titer, as demonstrated by Sanmiguel et al.[40] However, to the best of our knowledge, a comprehensive comparison involving other dPCR methods besides ddPCR has not been reported.

Capsid titer determination and handling times of capsid ELISA and BLI

The results shown in Figure 4 demonstrate the differences in capsid titer determination between the two methods BLI and ELISA. Although the relative SD for BLI was below 10%, it reached up to 16% for ELISA, which is at the upper boundary for this type of assay. Additionally, the linear measurement range for AAV8 capsid titer determination of ELISA was 1.5 log levels, which was 2.8-fold lower than the 4.2 log levels observed for BLI (Figures 4A and 4B). Consequently, BLI offers a much higher linear detection range. The detection range of both methods may vary slightly depending on the AAV serotype. On the other hand, ELISA is more sensitive and capable of detecting down to 8×10^6 c/mL, which is about 2.1 log levels lower than the BLI detection limit of 9 x 10^8 c/mL. The larger detection range of BLI offers the advantage that, when unknown samples are tested, one dilution is usually within the linear range. Furthermore, BLI is a serotype-independent measurement method because of the AAVX antibody coupled to the biosensor. In contrast, ELISA usually uses serotype-specific antibodies,[16] which in turn may also contribute to its increased sensitivity.

Significant differences ($\alpha = 0.05$, unpaired t test) between ELISA and BLI capsid titers were observed for AAV5 batch B and AAV8 batch A during analysis of AAV2, AAV5, and AAV8 samples (Figure 4C) from two different manufacturing processes. No significant differences were observed for the remaining samples ($\alpha = 0.05$, unpaired t test). Using the ELISA capsid titers as the reference, BLI capsid titers of AAV5 batch B and AAV8 batch A were found to be 112% and 130% of the corresponding ELISA values, respectively. In some samples, the capsid titers determined by BLI were higher than the ELISA

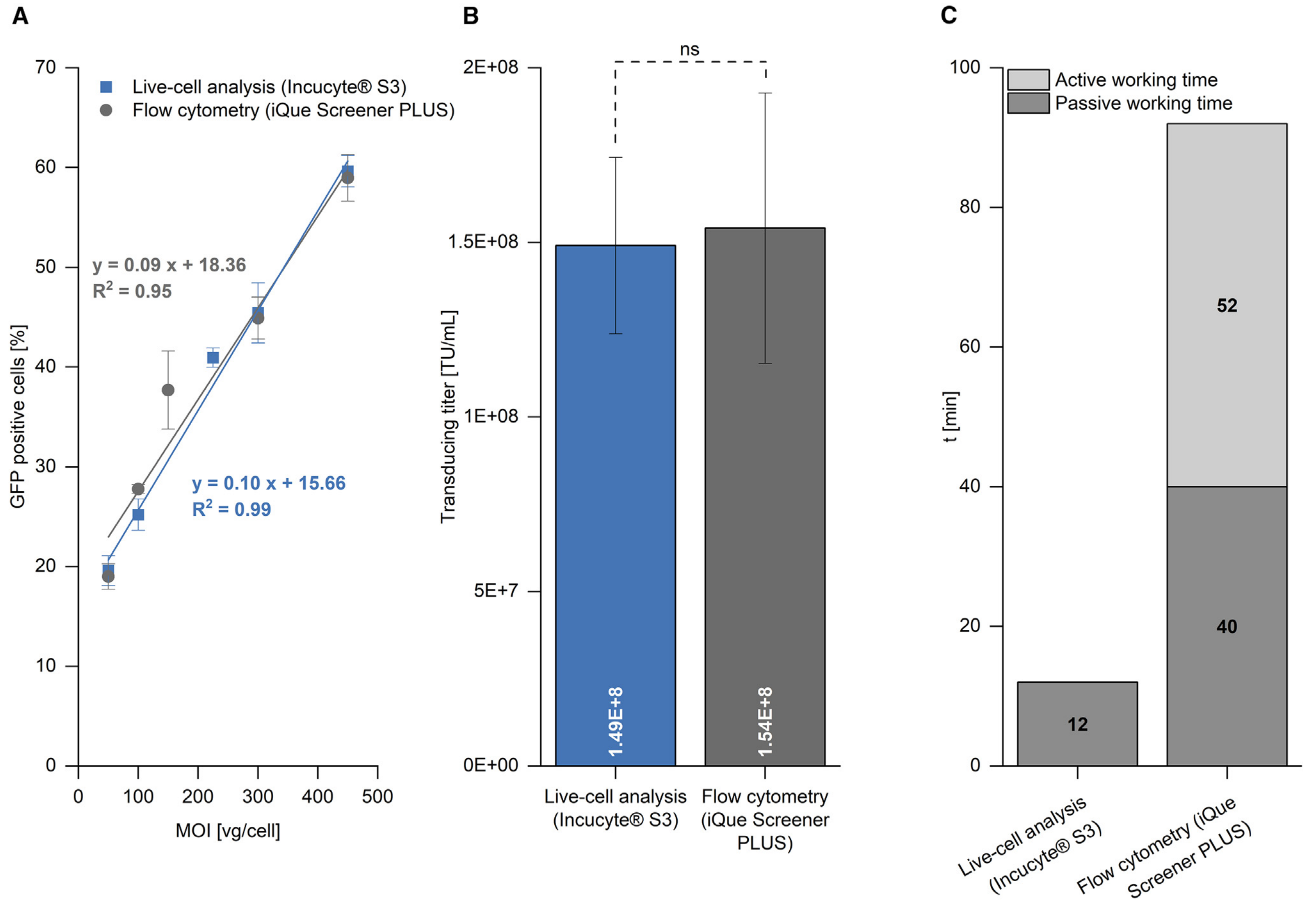

Figure 6. Transducing titer determination and handling times of live-cell analysis and flow cytometry

HEK293T cells were transduced with AAV2, and GFP expression was analyzed 48 h post-transfection by live-cell analysis (Incucyte S3) and flow cytometry (iQue Screener PLUS). A linear correlation between MOI used and the percentage of cells expressing GFP is shown, using values with a maximum relative SD of 10% (A). Furthermore, the determined transducing titer for an AAV2 sample by both methods is presented (B), and the active as well as the passive working time for both techniques for the processing of 32 samples is shown (C). The active working time describes manual handling steps, such as pipetting, whereas the passive working time refers to waiting times, such as incubation or measurement times. Procedures such as cell seeding and cultivation, transduction, and media exchange were identical for both methods and therefore not included in the working time analysis. Error bars represent SDs of triplicate measurements. Transducing titers were analyzed with an unpaired t test with a significance level of $\alpha = 0.05$. Non-significant differences are denoted by “ns.” TU, transducing units; MOI, multiplicity of infection; t, time.

capsid titers, and in other samples, lower than the ELISA capsid titers. Therefore, it is not possible to conclude that BLI or ELISA systematically leads to an overdetermination of the capsid titer or is influenced to a greater or lesser extent by the sample matrix. Especially, ELISA is known for its robustness toward matrix effects. According to the presented data, BLI demonstrates to be a viable alternative to the conventional ELISA technique for determining the capsid titer.

Analyzing the handling time of BLI and ELISA for the processing of 16 samples demonstrated considerable differences. Sample preparation and transfer to the 96-well plates were excluded from the active working analysis, as they were identical for both BLI and ELISA. For the remaining steps, BLI required an active working time of 10 min, while the ELISA method had an active working time of 42.5 min, which resulted from washing steps and the addition of further reagents required for the reaction. The passive working time was similar for both methods. BLI using the Octet R8 (Sartorius) had a passive working time of 72.3 min, whereas ELISA resulted in a passive working time of 86 min. In the BLI method, the passive working time is a singular uninterrupted block, whereas in the ELISA method, it is divided into multiple steps with intermittent hands-on periods. As the number of samples increases, the measurement time also rises in the BLI method, and so does the passive working time. However, by using the Octet RH96 instead of the Octet R8 used in this work, the measurement time could be considerably reduced, and thus improve the sample throughput. Alternatively, the active working time of ELISA could also be reduced by using automated liquid handling systems, such as pipetting robots. The BLI method on the Octet platform presents a more intriguing option for conducting routine analysis that requires high sample throughput and can be

easily scaled by selecting the appropriate instrument. When analyzing only a few samples on an occasional basis, the simplicity of the ELISA probably outweighs the cost of purchasing a BLI instrument. Numerous studies described the determination of the capsid titer using ELISA. Nevertheless, there is a lack of research on assessing the capsid titer by AAVX biosensors, which show the potential to streamline a high sample throughput while simultaneously minimizing the active working time.

Transducing titer determination and handling times of live-cell analysis and flow cytometry

A comparison of the two methods live-cell analysis and flow cytometry for the determination of the transducing titer revealed similar characteristics. The live-cell analysis exhibited a relative SD of $\leq$ 17%, while flow cytometry demonstrated a slightly higher relative SD of up to 25%. Moreover, both methods had an equal linear detection range between an MOI of 50–450 vg/cell (Figure 6A). The transducing titers determined using both methods in Figure 6B showed no statistically significant difference (α = 0.05, unpaired t test).

Analysis of the handling times for the transducing titer determination by live-cell analysis and flow cytometry revealed differences in their active and passive working times (Figure 6C). The live-cell analysis method required a shorter working time without active steps beyond the passive 96-well plate scan, while the flow cytometry protocol necessitated several active working steps with a longer overall working time. Live-cell analysis allows for increased sample throughput due to its reduced handling time, while the cytometry-based method is more sensitive. For the latter, the effort could be minimized by using suspension cells. Furthermore, if the flow cytometry is operated under high-throughput conditions, the impact of the 30 min purging of the device and calibration of the laser is reduced. However, gating of cell populations is operator dependent, which could possibly lead to variations between measurements performed by different operators. Recent studies have reported the suitability of live-cell analysis for determining the potency of lentivirus (LV) and vaccinia virus (VACV).[41,42] Live-cell analysis offers a major advantage by enabling temporal analysis, as the operator can determine the optimal analysis time point by tracking the transduced cells over time. In contrast, flow cytometry is an endpoint measurement that may miss the optimal time point for analysis. To the best of our knowledge, a comparison between live-cell analysis and flow cytometry for assessing the transducing titer of AAV has not been published yet. Moreover, the use of live-cell analysis for determination of the transducing titer of AAV has not been reported at all in any prior studies.

The HEK293-cell-based transduction assay used in this work is well suited for determining the transducing titer of AAV2 that encodes for a GFP gene. However, the transducing titers of AAV5 and AAV8 could not be determined through the transduction of HEK293 cells, as AAV5 and AAV8 bind to different cell receptors than AAV2.[43,44] As reported by Ellis et al., it is likely that the HEK293-cell-based transduction assay could also be used for AAV1 and AAV3, in addition to AAV2.[45] However, distinct cell lines are required for diverse AAV serotypes. The assay could also be adapted to AAVs that encode for a different transgene than GFP by using fluorescence *in situ* hybridization (FISH) probes or antibody-based fluorescent staining of target proteins expressed on the cell surface.

Conclusions

In this study, we compared standard analytical methods with more advanced methods for the determination of the VG titer, capsid titer, and transducing titer of AAV2, AAV5, and AAV8. The objective of this study was to establish and compare different methods with each other. In conclusion, we found that qPCR and four different dPCR methods resulted in comparable findings for purified samples. However, qPCR was more susceptible to matrix effects in unpurified samples, resulting in 1.4- to 2.6-fold higher VG titers. Furthermore, we demonstrated that Proteinase K treatment was not required for sample preparation of AAV2, AAV5, and AAV8. In addition, BLI emerged as a viable alternative to the commonly used ELISA for capsid titer determination, offering increased sample throughput and reduced labor time. The BLI approach provided a linear measurement range of 4.2 log levels, which is considerably larger than the 1.5 log levels of ELISA. For transducing titer determination, we developed a novel live-cell analysis assay and compared it with the commonly used flow cytometry. The determination of the transducing titer revealed no significant differences between flow cytometry and live-cell analysis, with the latter being less labor-intensive. In addition, both methods showed a similar linear measurement range, approximately between 19% and 59% GFP-positive cells.

MATERIALS AND METHODS

AAV production and harvest – Batch A

AAV2, AAV5, and AAV8 batch A were generated by transient transfection of HEK293 cells (Expi293F Inducible Cells, Thermo Fisher Scientific, Waltham, MA) in non-baffled glass shake flasks in FreeStyle 293 Expression Medium (Thermo Fisher Scientific). AAV production processes were inoculated at a cell density of 3×10^5 viable cells/mL. Upon achieving a cell density of 1.3×10^6 viable cells/mL, transfection was carried out using a two-plasmid system (PlasmidFactory, Bielefeld, Germany), with 1 μg/mL DNA per 10^6 viable cells and FectoVIR-AAV (Polyplus, Illkirch, France) as a transfection reagent in a 1:1 ratio. AAV vectors were harvested 72 h after transfection. For cell lysis, the cell broth was treated with Tween 20 (Sigma-Aldrich, Darmstadt, Germany), Denarase (c-Lecta, Leipzig, Germany), and $MgCl_2$ at final concentrations of 0.5%, 10 U/mL, and 2 mM, respectively, and incubated at 37°C for 1 h. Subsequently, the cell lysate was centrifuged at 800 × *g* for 5 min.

AAV production and harvest: Batch B

AAV2, AAV5, and AAV8 Batch B were generated by transient transfection of HEK293 cells (Expi293F Cells, Thermo Fisher Scientific) in 2 L bioreactor scale in HEK ViP NB Medium (Sartorius Xell, Schloß Holte-Stukenbrock, Germany). Cells were seeded at a cell density of 3×10^5 cells/mL on day 0 in a preculture/N-1 bioreactor. On day 3, AAV production processes were inoculated at a cell density of 2×10^6 viable cells/mL. Transient transfection was performed 24 h

after seeding using a two-plasmid system (PlasmidFactory), with 1 μg/mL DNA per 10^6 viable cells and FectoVIR-AAV as a transfection reagent in a 1:1 ratio. For cell lysis 72 h after transfection, the culture was continuously stirred (1 h, 37°C) after addition of a Tergitol TMN-100x-based lysis buffer (20 mM $MgCl_2$, 500 mM Tris, Tergitol TMN 1% [pH 7.5]) and Benzonase (both Sigma-Aldrich) at a final concentration of 25 U/mL. Subsequently, the cell lysate was centrifuged at 4,000 × g for 30 min.

Determination of genomic titers using qPCR and dPCR

For the determination of the VG titer, four different dPCR techniques and a qPCR approach were compared. AAV containing samples were serially diluted in dPCR buffer, which comprised TE buffer (Thermo Fisher Scientific), 0.01% Pluronic F-68 (Sigma-Aldrich), and 100 μg/mL Poly A Carrier RNA (Roche, Basel, Switzerland). The samples were subjected to incubation at 95°C for 30 min. For capsid lysis using Proteinase K (Minerva Biolabs, Berlin, Germany), the samples were incubated at 95°C for a duration of 15 min, and Proteinase K was added at a final concentration of 12 U/mL. The samples were incubated at 55°C for 60 min and then at 95°C for 15 min. dPCR and qPCR were performed as a duplex assay using specific forward and reverse primers (800 nM) and a specific probe (400 nM) that targeted the SV40 (Integrated DNA Technologies, Coralville, Iowa) with a FAM flurophore or the ITR (Microsynth, Balgach, Switzerland) region with a HEX fluorophore. Reaction mixes were prepared to comprise primers, probes, nuclease-free water and the respective mastermix. The following mastermixes were used: QIAcuity Probe Mastermix (Qiagen, Hilden, Germany), QuantStudio Absolute Q DNA Master Mix (Thermo Fisher Scientific), ddPCR Supermix for Probes (Bio-Rad, Hercules, California), naica multiplex PCR MIX (Stilla, Villejuif, France), and TaqMan Fast Advanced Master-Mix (Thermo Fisher Scientific).

ndPCR was conducted on the QIAcuity One using 24-well nanoplates with 26,000 cavities (Qiagen). Four microliters of extracted DNA sample was mixed with 36 μL reaction mix and transferred to the nanoplate, which was then sealed with a nanoplate seal (Qiagen). ndPCR was performed using a temperature profile consisting of 2 min at 95°C, followed by 40 cycles for 15 s at 95°C and for 30 s at 60°C. After 40 cycles, imaging was performed using the green and yellow channels with an exposure and gain time of 500 and 6 ms, respectively. The SD of independent triplicate measurements was less than 20%.

mapdPCR was performed on the QuantStudio Absolute Q Digital-PCR-System using 16-well microfluidic array plates (Thermo Fisher Scientific). One microliter of extracted DNA sample was mixed with 9 μL reaction mix and transferred to the microfluidic array plate. Fifteen microliters of isolation buffer (Thermo Fisher Scientific) was added to each well, and the plate was sealed with gaskets. mapdPCR was performed using a temperature profile consisting of 3 min at 95°C, followed by 40 cycles for 15 s at 95°C and for 30 s at 60°C. After completion of the 40 cycles, imaging was performed using the FAM and HEX channel. The SD of independent triplicate measurements was found to be below 15%.

ddPCR was performed on the QX200 Droplet Digital PCR System (Bio-Rad). Five point five microliters of extracted DNA sample was mixed with 16.5 μL reaction mix, transferred to a ddPCR 96-well plate (Bio-Rad), and sealed with a pierceable heat seal foil (Bio-Rad). Droplets were generated using the automated droplet generator (Bio-Rad). PCR was performed in a C1000 Touch Thermal Cycler (Bio-Rad) using a temperature profile consisting of 10 min at 95°C, followed by 40 cycles for 30 s at 94°C and for 60 s at 60°C, and a final polymerase inactivation step of 10 min at 98°C. After completion of the 40 cycles, the fluorescence intensity of the droplets was measured with the QX200 Droplet Reader (Bio-Rad) using the FAM and HEX channel. The SD of independent triplicate measurements was ≤14%.

cdPCR was performed on the naica system using ruby chips (Stilla). One microliter of extracted DNA sample were mixed with 4 μL reaction mix and transferred to a ruby chip, which was subsequently wiped with an anti-static cloth. cdPCR was performed using a temperature profile consisting of 3 min at 95°C, followed by 40 cycles for 15 s at 95°C and for 30 s at 60°C. After completion of the 40 cycles, imaging was performed using the FAM and HEX channels with an exposure time of 65 and 250 ms, respectively. The SD of independent triplicate measurements was less than 13%.

qPCR was conducted using the CFX96 Deep Well Real-Time PCR System (Bio-Rad) and the vector plasmid pAAV-ssGFP (PlasmidFactory) as a standard. The concentration of the plasmid was determined using the manufacturer-provided DNA concentration of the vector plasmid and its molecular weight. Linearization of the plasmid was performed using 10 U/mL Eco105I (Thermo Fisher Scientific) at 37°C for 20 min, followed by a step at 65°C for 20 min. The expected concentration of the linearized plasmid was verified by dPCR measurements. Two microliters of extracted DNA sample were mixed with 18 μL reaction mix. qPCR was performed using a temperature profile consisting of 2 min at 50°C and 2 min at 95°C, followed by 40 cycles for 15 s at 95°C and for 30 s at 60°C. After each cycle, the fluorescence signal was measured using the FAM and HEX channel. VG copies were quantified against a linear fitted standard curve in the range of 10^1 to 10^6 copies/μL. Results were obtained from independent triplicate measurements with an SD of ≤69%.

Determination of capsid titers using ELISA and BLI

ELISA: capsid titers were determined using the AAV Xpress ELISA kits (Progen Biotechnik, Heidelberg, Germany) following the manufacturer's instructions. Capsid titers were calculated using a linear fitted standard curve. Results were obtained from duplicate measurements with an SD of less than 16%.

BLI: experiments were conducted using the Octet R8 instrument, in conjunction with Octet AAVX Biosensors (Sartorius, Göttingen, Germany). The final three columns of the microplate were used for washing of the biosensors in assay buffer (Octet Sample Diluent, Sartorius), regeneration in 10 mM glycine buffer (pH 1.7), and neutralization in assay buffer. Quantitation was performed at a temperature of 30°C and a shaking speed of 1,000 rpm. The quantitation

step reading time was set to 900 s. Following the measurement of the sample, the biosensors were washed for 180 s and subjected to a 5×5 s regeneration and neutralization step and re-used. Titer determination was accomplished using a 4-parameter logistic (4PL) weighted Y-fitted standard curve. AAV reference standards, including AAV2 and AAV5 obtained from Progen Biotechnik, along with self-purified AAV8, were used to generate the standard curve. The SD of triplicate measurements was found to be below 10%.

Determination of transducing titers using flow cytometry and live-cell analysis

For determination of the transducing titer, adherent HEK293T cells (ACC 635; DSMZ, Braunschweig, Germany) were transduced with AAV2 samples. Per well, 4×10^3 HEK293 cells were seeded in DMEM (Thermo Fisher Scientific) supplemented with 10% (v/v) fetal calf serum (FCS) and 0.5% (v/v) penicillin-streptomycin (P/S) (both Sigma-Aldrich) in a tissue culture (TC) treated, poly-L-lysine (Sigma-Aldrich) coated black 96-well plate with clear bottom (Corning Inc., Corning, New York). Cells were cultivated for one day at 36.5°C and 5% CO_2 in a static incubator. For transduction, the spent culture medium was removed and cells were transduced by adding 50 μL of serially diluted AAV2 samples. Twenty hours after transduction, the AAV2 containing samples were removed from the wells and replaced with 50 μL fresh DMEM (plus FCS and P/S). Forty-eight hours post-transduction, the expression of GFP was analyzed using either flow cytometry or live-cell analysis.

Flow cytometry: for GFP expression analysis, cells were detached from the culture plate by incubating with 20 μL trypsin-EDTA (Thermo Fisher Scientific) for 5 min at 37°C. The trypsin reaction was terminated by adding 30 μL DMEM, and the detached cells were transferred to a non-TC-treated 96-well plate with conical bottom (Sartorius). Following detachment, the cells were washed with PBS and fixed with 100 μL of Roti-Histofix 10% (Carl Roth, Karlsruhe, Germany) for 15 min. Subsequently, the cells were washed with PBS and resuspended in 40 μL PBS. Flow cytometry was performed on the iQue Screener Plus (Sartorius). The transducing titers (given in TU per milliliter) were calculated using equation 1, in which N represents the number of cells at transduction, F corresponds to the percentage of GFP expressing single cells, D signifies the dilution factor of the AAV sample, and V indicates the volume of transduction. Results were obtained from triplicate measurements within the linear detection range with an SD of ≤25%.

Live-cell analysis: the 96-well plate was placed in the Incucyte® S3 (Sartorius) immediately after cell seeding, which was located in a static incubator. At intervals of 3 h, each well of the 96-well plate was imaged with 4 images at 10× magnification, using both the phase contrast channel and the green fluorescence channel. The Incucyte software was used to analyze the area of cells expressing GFP and the confluent area. The ratio of these two areas was used to determine the percentage of cells expressing GFP. Therefore, the phase segmentation was set to 1.2, the minimum area to 170 μm^2, and the cleanup to 1 pixel. GFP analysis was achieved by using a top-hat segmentation with deactivated edge split off, a threshold of 0.4 green calibrated units, a cleanup of 3 pixels, and a minimum area of 35 μm^2. Furthermore, the number of cells at transduction was determined by correlating the confluent area with offline cell counts using Cedex HiRes analyzer (Roche) as reported by Labisch et al.[41] The transducing titers were calculated using formula 1. The SD of triplicate measurements within the linear detection range was ≤17%.

$$\text{Infectious titer} = \frac{N * F * D}{V * 100} \qquad \text{(Equation 1)}$$

DATA AND CODE AVAILABILITY

Data will be made available on request.

SUPPLEMENTAL INFORMATION

Supplemental information can be found online at https://doi.org/10.1016/j.omtm.2023.101118.

ACKNOWLEDGMENTS

This publication is part of a cumulative PhD at the Graduate Centre for Engineering Sciences/Promotionszentrum für Ingenieurswissenschaften under the aegis of the University of Applied Sciences Mittelhessen, Giessen, Germany. The work was funded by Sartorius Lab Instruments GmbH & Co. KG (Göttingen, Germany). This research did not receive any specific grant from funding agencies in the public, commercial, or not-for-profit sectors. The authors thank Robert Hertel, Katja Heinrichs, Bob Dass, Jennifer Labisch, and Niklas Krämer for their guidance on using new analytics devices and fruitful discussions. Furthermore, the authors would like to thank Sandra Klausing, Alyssa Vetter, and Kathrin Teschner for providing AAV material from a different upstream process.

AUTHOR CONTRIBUTIONS

F.M., conceptualization, methodology, investigation, formal analysis, visualization, and writing – original draft. A.K., methodology and investigation. M.P., methodology and investigation. K.P., supervision. M.W.W., supervision and writing – review & editing. A.P., supervision, writing – review & editing, and project administration.

DECLARATION OF INTERESTS

The authors declare no competing interests.

DECLARATION OF GENERATIVE AI IN SCIENTIFIC WRITING

During the preparation of this work the authors used DeepL Translate, DeepL Write, and Chat GPT in order to improve readability and language. After using the tools, the authors reviewed and edited the content as needed and take full responsibility for the content of the publication.

REFERENCES

1. Bin Umair, M., Akusa, F.N., Kashif, H., Seerat-E-Fatima, Butt, F., Azhar, M., Munir, I., Ahmed, M., Khalil, W., Sharyar, H., et al. (2022). Viruses as tools in gene therapy, vaccine development, and cancer treatment. Arch. Virol. *167*, 1387–1404.

2. Dhungel, B.P., Bailey, C.G., and Rasko, J.E.J. (2021). Journey to the Center of the Cell: Tracing the Path of AAV Transduction. Trends Mol. Med. *27*, 172–184.

3. Berns, K.I., and Muzyczka, N. (2017). AAV: An Overview of Unanswered Questions. Hum. Gene Ther. *28*, 308–313.

4. John Wileys and Sons (2023). Gene Therapy Clinical Trials Worldwide. J. Gene Med.

5. Atchison, R.W., Casto, B.C., and Hammon, W.M. (1965). Adenovirus-associated Defective Virus Particles, *149*, pp. 754–756.

6. Ayuso, E. (2016). Manufacturing of Recombinant Adeno-Associated Viral Vectors: New Technologies Are Welcome, *3*, 15049.

7. Galibert, L., and Merten, O.-W. (2011). Latest developments in the large-scale production of adeno-associated virus vectors in insect cells toward the treatment of neuromuscular diseases. J. Invertebr. Pathol. *107* (*Suppl*), S80–S93.

8. Verdera, H.C., Kuranda, K., and Mingozzi, F. (2020). AAV Vector Immunogenicity in Humans: A Long Journey to Successful Gene Transfer. Mol. Ther. *28*, 723–746.

9. Srivastava, A. (2016). In vivo tissue-tropism of adeno-associated viral vectors. Curr. Opin. Virol. *21*, 75–80.

10. Grieger, J.C., and Samulski, R.J. (2012). Adeno-associated virus vectorology, manufacturing, and clinical applications. Methods Enzymol. *507*, 229–254.

11. Beloukhova, M.I., Lukashev, A.N., Volchkov, P.Y., Zamyatnin, A.A., and Deviatkin, A.A. (2022). Robust AAV Genotyping Based on Genetic Distances in Rep Gene That Are Maintained by Ubiquitous Recombination. Viruses *14*.

12. Weinberg, M.S., Samulski, R.J., and McCown, T.J. (2013). Adeno-associated virus (AAV) gene therapy for neurological disease. Neuropharmacology *69*, 82–88.

13. Arnold, G.S., Sasser, A.K., Stachler, M.D., and Bartlett, J.S. (2006). Metabolic biotinylation provides a unique platform for the purification and targeting of multiple AAV vector serotypes. Mol. Ther. *14*, 97–106. 1525-0016.

14. Blessing, D., Vachey, G., Pythoud, C., Rey, M., Padrun, V., Wurm, F.M., Schneider, B.L., and Déglon, N. (2019). Scalable Production of AAV Vectors in Orbitally Shaken HEK293 Cells, *13*, pp. 14–26, 2329-0501.

15. U.S. Food & Drug Administration (2009). Guidance for Industry: Q8(R2) (Pharmaceutical Development).

16. Tustian, A.D., and Bak, H. (2021). Assessment of quality attributes for adeno-associated viral vectors. Biotechnol. Bioeng. *118*, 4186–4203.

17. Lock, M., Alvira, M.R., Chen, S.-J., and Wilson, J.M. (2014). Absolute determination of single-stranded and self-complementary adeno-associated viral vector genome titers by droplet digital PCR. Hum. Gene Ther. Methods *25*, 115–125.

18. Martinez-Fernandez de la Camara, C., McClements, M.E., and MacLaren, R.E. (2021). Accurate Quantification of AAV Vector Genomes by Quantitative PCR. Genes *12*, 601.

19. Wolff, J., Beer, M., and Hoffmann, B. (2021). Probe-Based Real-Time qPCR Assays for a Reliable Differentiation of Capripox Virus Species. Microorganisms *9*, 765.

20. Taylor, S.C., Laperriere, G., and Germain, H. (2017). Droplet Digital PCR versus qPCR for gene expression analysis with low abundant targets: from variable nonsense to publication quality data. Sci. Rep. *7*, 2409.

21. Dorange, F., and Le Bec, C. (2018). Analytical approaches to characterize AAV vector production & purification: Advances and challenges. Cell Gene Therapy Insights *4*, 119–129.

22. Gimpel, A.L., Katsikis, G., Sha, S., Maloney, A.J., Hong, M.S., Nguyen, T.N.T., Wolfrum, J., Springs, S.L., Sinskey, A.J., Manalis, S.R., et al. (2021). Analytical Methods for Process and Product Characterization of Recombinant Adeno-Associated Virus-Based Gene Therapies, *20*, pp. 740–754, 2329-0501.

23. Ramsey, J.P., Khatwani, S.L., Lin, M., Boregowda, R., Surosky, R., and Andrew Ramelmeier, R. (2021). Overview of analytics needed to support a robust gene therapy manufacturing process. Current Opinion in Biomedical Engineering *20*, 100339.

24. Shah, N.B., and Duncan, T.M. (2014). Bio-layer interferometry for measuring kinetics of protein-protein interactions and allosteric ligand effects. J. Vis. Exp. e51383.

25. Müller-Esparza, H., Osorio-Valeriano, M., Steube, N., Thanbichler, M., and Randau, L. (2020). Bio-Layer Interferometry Analysis of the Target Binding Activity of CRISPR-Cas Effector Complexes. Front. Mol. Biosci. *7*, 98.

26. François, A., Bouzelha, M., Lecomte, E., Broucque, F., Penaud-Budloo, M., Adjali, O., Moullier, P., Blouin, V., and Ayuso, E. (2018). Accurate Titration of Infectious AAV Particles Requires Measurement of Biologically Active Vector Genomes and Suitable Controls, *10*, pp. 223–236.

27. Zeltner, N., Kohlbrenner, E., Clément, N., Weber, T., and Linden, R.M. (2010). Near-perfect infectivity of wild-type AAV as benchmark for infectivity of recombinant AAV vectors. Gene Ther. *17*, 872–879.

28. Lothert, K., Eilts, F., and Wolff, M.W. (2022). Quantification methods for viruses and virus-like particles applied in biopharmaceutical production processes. Expert Rev. Vaccines *21*, 1029–1044.

29. Ettinger, A., and Wittmann, T. (2014). Fluorescence live cell imaging. Methods Cell Biol. *123*, 77–94.

30. Edlund, C., Jackson, T.R., Khalid, N., Bevan, N., Dale, T., Dengel, A., Ahmed, S., Trygg, J., and Sjögren, R. (2021). LIVECell-A large-scale dataset for label-free live cell segmentation. Nat. Methods *18*, 1038–1045.

31. Ye, M., Wilhelm, M., Gentschev, I., and Szalay, A. (2021). A Modified Limiting Dilution Method for Monoclonal Stable Cell Line Selection Using a Real-Time Fluorescence Imaging System: A Practical Workflow and Advanced Applications. Methods Protoc. *4*, 16.

32. Wang, D., Tai, P.W.L., and Gao, G. (2019). Adeno-associated virus vector as a platform for gene therapy delivery. Nat. Rev. Drug Discov. *18*, 358–378.

33. Hebben, M. (2018). Downstream bioprocessing of AAV vectors: industrial challenges & regulatory requirements. Cell Gene Therapy Insights *4*, 131–146.

34. Furuta-Hanawa, B., Yamaguchi, T., and Uchida, E. (2019). Two-Dimensional Droplet Digital PCR as a Tool for Titration and Integrity Evaluation of Recombinant Adeno-Associated Viral Vectors. Hum. Gene Ther. Methods *30*, 127–136.

35. D'Costa, S., Blouin, V., Broucque, F., Penaud-Budloo, M., François, A., Perez, I.C., Le Bec, C., Moullier, P., Snyder, R.O., and Ayuso, E. (2016). Practical utilization of recombinant AAV vector reference standards: focus on vector genomes titration by free ITR qPCR. Mol. Ther. Methods Clin. Dev. *5*, 16019.

36. Cankar, K., Stebih, D., Dreo, T., Zel, J., and Gruden, K. (2006). Critical points of DNA quantification by real-time PCR–effects of DNA extraction method and sample matrix on quantification of genetically modified organisms. BMC Biotechnol. *6*, 37.

37. Dobnik, D., Kogovšek, P., Jakomin, T., Košir, N., Tušek Žnidarič, M., Leskovec, M., Kaminsky, S.M., Mostrom, J., Lee, H., and Ravnikar, M. (2019). Accurate Quantification and Characterization of Adeno-Associated Viral Vectors. Front. Microbiol. *10*, 1570.

38. Rayaprolu, V., Kruse, S., Kant, R., Venkatakrishnan, B., Movahed, N., Brooke, D., Lins, B., Bennett, A., Potter, T., McKenna, R., et al. (2013). Comparative analysis of adeno-associated virus capsid stability and dynamics. J. Virol. *87*, 13150–13160.

39. Bennett, A., Patel, S., Mietzsch, M., Jose, A., Lins-Austin, B., Yu, J.C., Bothner, B., McKenna, R., and Agbandje-McKenna, M. (2017). Thermal Stability as a Determinant of AAV Serotype Identity, *6*, pp. 171–182, 2329-0501.

40. Sanmiguel, J., Gao, G., and Vandenberghe, L.H. (2019). Quantitative and Digital Droplet-Based AAV Genome Titration. Methods Mol. Biol. *1950*, 51–83.

41. Labisch, J.J., Wiese, G.P., Barnes, K., Bollmann, F., and Pflanz, K. (2021). Infectious titer determination of lentiviral vectors using a temporal immunological real-time imaging approach. PLoS One *16*, e0254739.

42. Ye, M., Keicher, M., Gentschev, I., and Szalay, A.A. (2021). Efficient Selection of Recombinant Fluorescent Vaccinia Virus Strains and Rapid Virus Titer Determination by Using a Multi-Well Plate Imaging System. Biomedicines *9*, 1032.

43. Summerford, C., and Samulski, R.J. (1998). Membrane-associated heparan sulfate proteoglycan is a receptor for adeno-associated virus type 2 virions. J. Virol. *72*, 1438–1445. 0022-538X.

44. Walters, R.W., Yi, S.M., Keshavjee, S., Brown, K.E., Welsh, M.J., Chiorini, J.A., and Zabner, J. (2001). Binding of adeno-associated virus type 5 to 2,3-linked sialic acid is required for gene transfer. J. Biol. Chem. *276*, 20610–20616.

45. Ellis, B.L., Hirsch, M.L., Barker, J.C., Connelly, J.P., Steininger, R.J., and Porteus, M.H. (2013). A survey of ex vivo/in vitro transduction efficiency of mammalian primary cells and cell lines with Nine natural adeno-associated virus (AAV1-9) and one engineered adeno-associated virus serotype. Virol. J. *10*, 74.

OMTM, Volume *31*

Supplemental information

Unveiling the secrets of adeno-associated virus: novel high-throughput approaches for the quantification of multiple serotypes

Frederik Meierrieks, Ahmad Kour, Marvin Pätz, Karl Pflanz, Michael W. Wolff, and Andreas Pickl

Table S1: Cost estimation per sample for each method. The costs per data point are calculated based on the respective consumable costs and available sample positions. Expenses related to instrument costs and salaries were excluded from the estimation process. Consumable expenses for each method were derived from the information available on the German manufacturer's website. It should be noted that prices vary between countries and depending on the order quantity. Therefore, the table provides only a rough overview of the costs per data point.

	Method	Price per data point [€]
Genome titer	ndPCR (Qiagen)	3-4
	mapdPCR (Thermo Fisher)	6-7
	cdPCR (Stilla)	3-4
	ddPCR (Bio-Rad)	1-2
	qPCR (Bio-Rad)	1-2
Capsid titer	Capsid ELISA	8-9
	BLI (Octet®)	5-6
Transducing titer	Live-cell analysis (Incucyte® S3)	~1
	Flow cytometry (iQue Screener PLUS)	~1

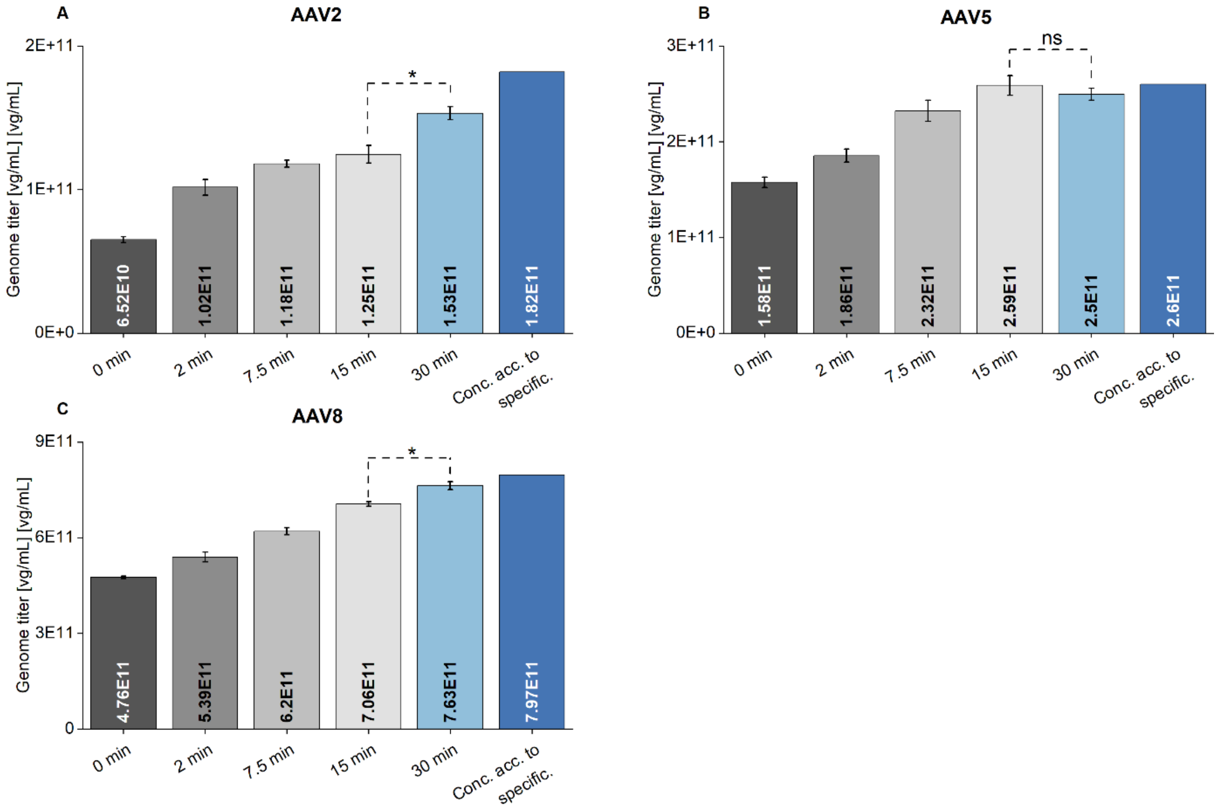

Figure S1: Impact of the heat incubation on AAV capsid disassembly. AAV2, AAV5, and AAV8 reference standard materials (AMSBIO Europe, Alkmaar, The Netherlands) were incubated at 95°C for different times prior to a 2 min initial denaturation at 95°C during PCR. The VG titers of AAV2 (**A**), AAV5 (**B**), and AAV8 (**C**) reference standard materials were determined by ndPCR (Qiagen) targeting the SV40 signal on the AAV genome. Error bars represent standard deviations of independent triplicate measurements. Statistical analysis was performed by unpaired t-test with a significance level of α=0.05. Statistically significant difference is denoted by an asterisk (*), whereas "ns" indicates no significant difference. Abbreviations: vg, viral genome.
